# Supplementary material for: DMRforPairs: identifying Differentially Methylated Regions between unique samples using array based methylation profiles
Source: BMC Bioinformatics. 2014 May 15;15:141. doi: 10.1186/1471-2105-15-141 (PMC4046028; doi:10.1186/1471-2105-15-141)
Supplement: Additional file 2 — DMRforPairs output for the comparison of A431-MCF7 and NA17018-NA17105. Please start from the HTML files in each folder. Available via the BMC Bioinformatics website. [file 1471-2105-15-141-S2.zip › 1394847754114233_MOESM2_ESM/A431_MCF7/figures/39782.pdf]

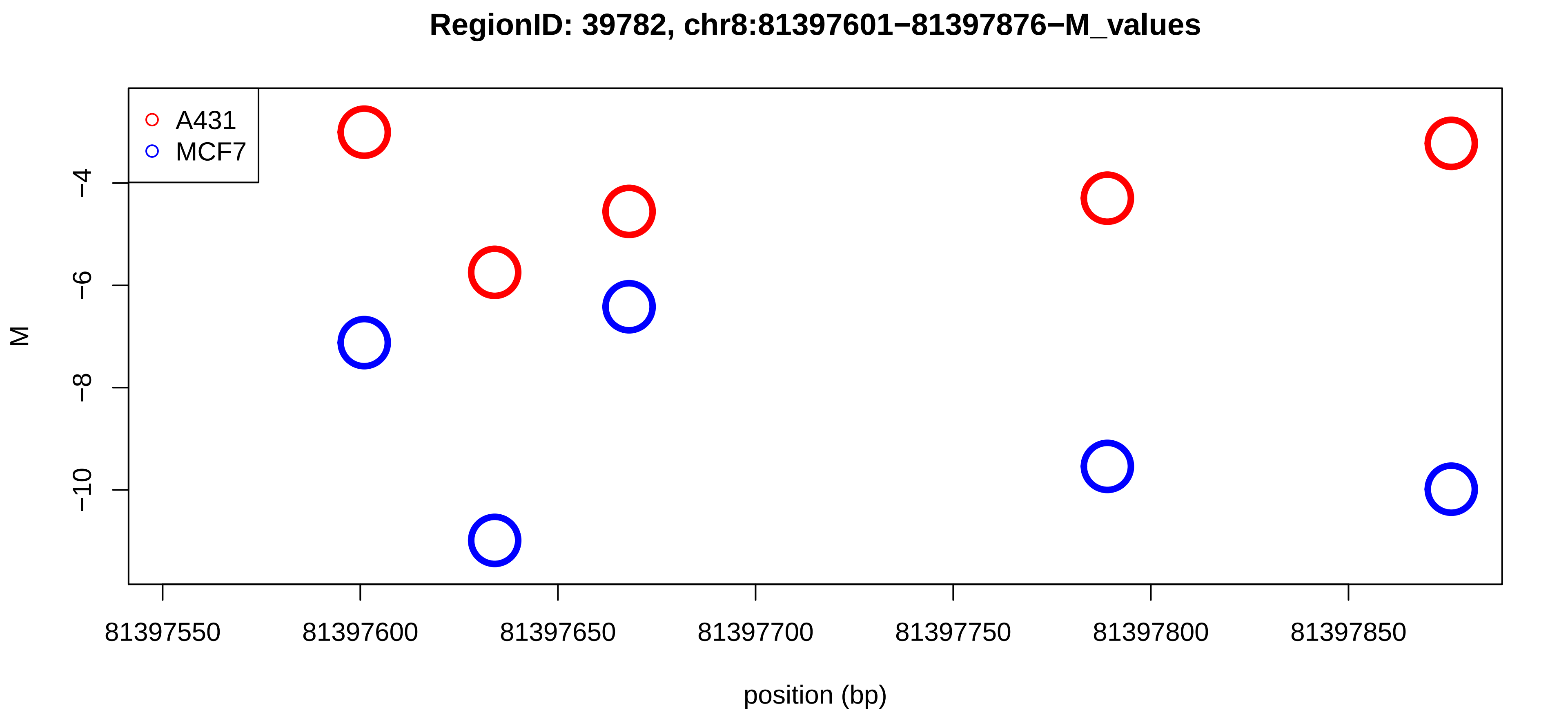
RegionID: 39782, chr8:81397601−81397876−M_values
A431
MCF7
M
−4
−6
−8
−10
81397550
81397600
81397650
81397700
81397750
81397800
81397850
position (bp)

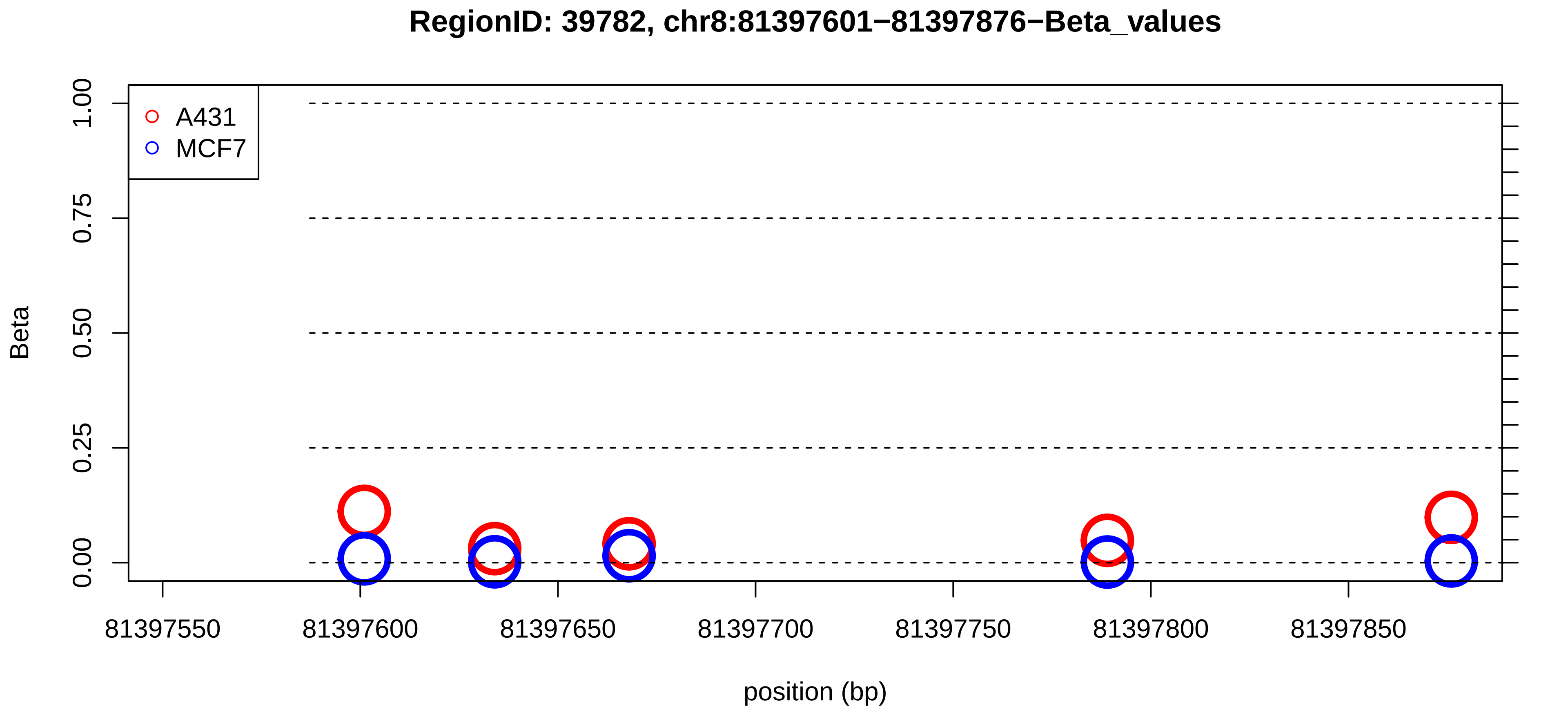
RegionID: 39782, chr8:81397601−81397876−Beta_values
A431
MCF7
Beta
1.00
0.75
0.50
0.25
0.00
81397550
81397600
81397650
81397700
81397750
81397800
81397850
position (bp)

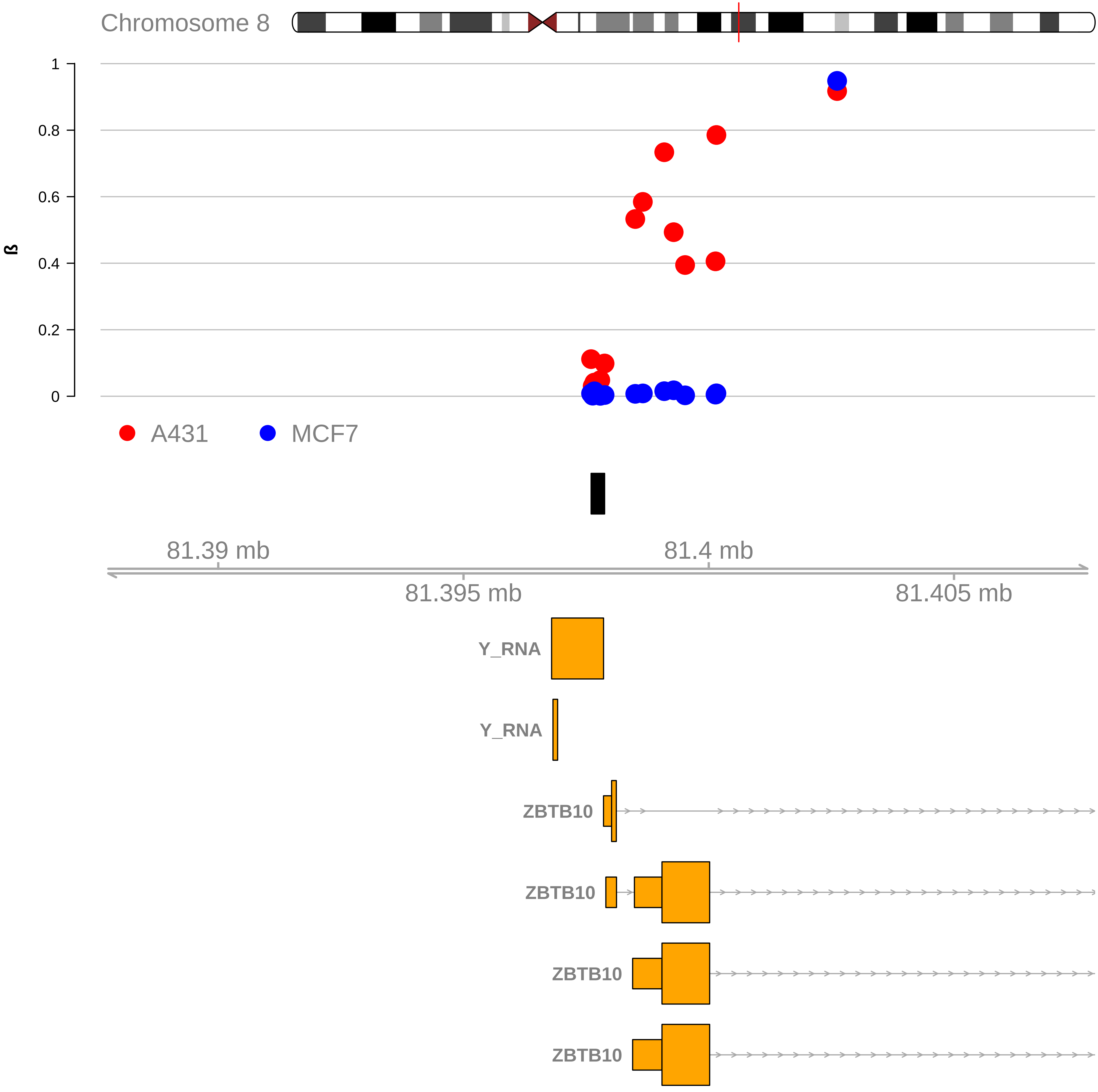
Chromosome 8
1
0.8
0.6
0.4
0.2
0
ß
A431
MCF7
81.39 mb
81.4 mb
81.395 mb
81.405 mb
Y_RNA
Y_RNA
ZBTB10
ZBTB10
ZBTB10
ZBTB10
